# Supplementary material for: Feasibility and results of joint ambulatory monitoring of exposure to particulate matter pollution and lung function in children in Abidjan, Côte d’Ivoire: a cross-sectional observational study
Source: BMJ Open. 2026 Feb 15;16(2):e109615. doi: 10.1136/bmjopen-2025-109615 (PMC12927363; doi:10.1136/bmjopen-2025-109615)
Supplement: Supplementary file 2 [file bmjopen-2025-109615.reviewer_comments.pdf]

PEER REVIEW HISTORY

BMJ Open publishes all reviews undertaken for accepted manuscripts. Reviewers are asked to complete a checklist review form and are provided with free text boxes to elaborate on their assessment. These free text comments are reproduced below.

ARTICLE DETAILS

Title (Provisional)

Feasibility and results of joint ambulatory monitoring of exposure to particulate matter pollution and lung function in children in Abidjan, Côte d'Ivoire, a cross-sectional observational study.

Authors

Pajot, Auriane; Yapo, Marie; Liousse, Catherine; Doumbia, Madina; Gnamien, Sylvain; Ahoua, Stéphane; Djé, Sonia; Dick, Flore; Fayon, Michael; Yoboue, Véronique; Marcy, Olivier

---

VERSION 1 - REVIEW

**Reviewer**	**1**
**Name**	**Lu, Chan**
**Affiliation**	**Central South University, XiangYa School of Public Health**
**Date**	**06-Sep-2025**
**COI**	**None**

This manuscript is relatively new and has a great meaning. It is a cross-sectional study assessing the feasibility and health impacts of joint ambulatory PM2.5 and lung function monitoring among children in Abidjan, Côte d'Ivoire. It reports high pollution levels and short-term lung function decline post-exposure. This article is well designed and scientifically written. However, there are some problems in this manuscript.

Due to the great meaning and a possibility in an improvement after a major revision, I suggest the editor accept this manuscript with major revision.

I have some comments and suggestions for this manuscript as follows:

1. Small sample size.

Only 29 children were included, and although this is a feasibility study, the possible limitations of insufficient statistical validity need to be explicitly accounted for, especially in stratified analyses and multivariate models.

2. Data validity.

Only 28.4% of lung function curves met the ATS/ERS criteria, further discussion is needed on the difficulty of the technical maneuver, the reasons for the low level of cooperation of the children, and how to improve the quality of the data in the future (e.g., equipment improvement, training reinforcement).

3. PM2.5 Sensor calibration.

Although the use of group-specific calibration factors is mentioned, the validation process of the calibration method is not described in detail, and it is recommended that additional sensor accuracy validation data be provided or that relevant literature be cited.

4. Environmental covariates were missing.

Factors such as temperature and humidity that may affect lung function were not controlled for, and it is recommended that this limitation be clarified in the discussion and that sensitivity analyses be performed when possible.

5. The discussion section could be deepened.

It is recommended that the discussion on the public health implications of the results and policy recommendations be strengthened, in particular the urgency of protective measures for children in high-exposure environments.

6. The authors mentioned that "High PM2.5 exposure and individual variability in lung function highlight the need for further research respiratory effects of air pollution in children", but this conclusion is obtained from the participants' current observation. Some recent studies indicate that early-life exposure to air pollution especially traffic-related air pollution (TRAP) including PM2.5 and NO2 is associated with childhood respiratory diseases (e.g., pneumonia and COVID-19). Some other new studies explain the potential mechanisms underlying the associations between TRAP (e.g., NO2) and allergic asthma, such as oxidative stress (e.g., ROS) and airway inflammation. I suggestion the authors should add some evidence in the effects of air pollution exposure on lung function. Please discuss your findings with (some of) these new articles to further support your results and explain the potential mechanisms.

[1] https://doi.org/10.1016/j.jhazmat.2023.131787

[2] https://doi.org/10.1016/j.jhazmat.2023.131837

[3] https://doi.org/10.1016/j.jhazmat.2025.137940

Reviewer **2**

Name **Sommar, Johan**

Affiliation **Umea University Department of Public Health and Clinical Medicine, Sustainable health**

Date **21-Oct-2025**

COI None

Thank you for giving me the opportunity to read the manuscript bmjopen-2025-109615, entitled "Feasibility and results of joint ambulatory monitoring of exposure to particulate matter pollution and lung function in children in Abidjan, Côte d'Ivoire, a cross-sectional observational study". This is indeed an interesting and important study on the feasibility of monitoring of exposure to fine particulate matter ($PM_{2.5}$) and spirometry in children living in a low- to mid-income country. A strength of the study is that it is based on measurements and not modelled concentrations. The study would have had scientific value also without assessments of respiratory outcomes.

The study however assesses such associations using state-of-the-art statistical methods able to capture also delayed effects and thereby identify important time windows of exposure and presenting cumulative effects of exposure.

Results are presented in an excellent way.

I would however like the authors to evaluate if there is any empirical support for non-linear associations between ppFEV1 and PM2.5. There is no mention of whether this was assessed. Also, the assessment of other model assumptions is lacking. Was for instance the assumptions of constant variance reasonably fulfilled?

This seems to be a gross misinterpretation of the result provided by the model:

"Linking $ppFEV_1$ with $PM_{2.5}$ exposure revealed a transient decline in lung function, with the strongest effect observed few hours after an increase in $PM_{2.5}$ levels equivalent to the interquartile range of daily exposure."

VERSION 1 - AUTHOR RESPONSE

Reviewer 1

We thank Reviewer 1 for the interest and time accorded to the revision of our article. We addressed their questions and comments in a point-by-point response below.

In addition, we have taken this opportunity for edits to improve the writing that are in track change in the manuscript. We hope that this has also contributed to improve the overall quality of our manuscript.

Reviewer comments

1. Small sample size. Only 29 children were included, and although this is a feasibility study, the possible limitations of insufficient statistical validity need to be explicitly accounted for, especially in stratified analyses and multivariate models.

Authors' response

We agree with the reviewer that the small sample size is a limitation of our study. As this was a feasibility study, our primary objective was to assess the practicality of joint ambulatory monitoring of PM exposure and lung function among children, rather than to achieve statistical representativeness.

Nevertheless, we have now explicitly acknowledged the limited statistical power in the revised Limitations section (Discussion). We have clarified that the results should be interpreted with caution and mainly as indicative trends that justify larger-scale studies in the future.

"This study has several limitations. The small sample size and short duration (seven days) limit the ability to capture long-term variability. A limited number of valid spirometry curves may have reduced statistical power and representativeness. The results should be interpreted with caution and mainly as indicative trends that justify larger-scale studies in the future."

Reviewer comments

2. Data validity. Only 28.4% of lung function curves met the ATS/ERS criteria, further discussion is needed on the difficulty of the technical maneuver, the reasons for the low level of cooperation of the children, and how to improve the quality of the data in the future (e.g., equipment improvement, training reinforcement).

Authors' response
We thank the reviewer for this helpful comment. We agree that the relatively low proportion of spirometry curves meeting ATS/ERS validity criteria represents an important limitation. We have now expanded the Discussion to provide a more detailed explanation.

Specifically, we discuss that the technical maneuver is challenging for children in ambulatory conditions and that the absence of real-time feedback or motivational features in the spirometers likely contributed to suboptimal cooperation.

We have also emphasized that future studies could improve data validity through enhanced participant training, repeated practice sessions, and the use of more child-friendly or interactive devices that provide visual feedback to encourage maximal effort.

This additional discussion clarifies that while our results confirm the feasibility of ambulatory spirometry, optimization of data quality remains essential.

"A high proportion of volume flow curves was collected, reflecting good engagement and feasibility of repeated ambulatory spirometry on children. However, fewer than a third of the curves met ATS/ERS validity criteria, highlighting technical limitations and challenges related

to children's cooperation during unsupervised maneuvers. The limited participant training, and the absence of repeated supervised practice and feedback or motivational features in the devices used may have reduced performance quality. Previous studies have shown that child-friendly interactive tools can improve spirometry quality (26), such techniques may be applied in future studies ."

Reviewer comments

3. $PM_{2.5}$ Sensor calibration. Although the use of group-specific calibration factors is mentioned, the validation process of the calibration method is not described in detail, and it is recommended that additional sensor accuracy validation data be provided or that relevant literature be cited.

Authors' response

We thank the reviewer for this valuable comment. We agree that further clarification of the calibration and validation process was needed. We have therefore expanded the Methods section to describe how the $PM_{2.5}$ calibration factors were obtained and applied. Specifically, we now indicate that $PM_{2.5}$ concentrations were corrected using group-specific calibration factors, which were derived from gravimetric reference measurements performed on filters in the same study neighborhoods. These gravimetric measurements were conducted within the framework of the APIMAMA research project and were used to calibrate and validate the portable optical sensors used in this study.

A new reference has also been added to support the calibration approach based on gravimetric data (Long-term field evaluation of the Plantower PMS low-cost particulate matter sensors, Sayahi et al. 2019). (https://doi-org.docelec.u-bordeaux.fr/10.1016/j.envpol.2018.11.065) The detailed results of the gravimetric calibration will be reported in a separate forthcoming article.

"$PM_{2.5}$ concentrations were corrected using group-specific calibration factors (5.5 for Groups 1 and 3, and 5.0 for Group 2) (25). Gravimetric measurements performed on filters, were used to calibrate and validate the optical particle sensors (26). Concentrations below 6 µg/m³, corresponding to the sensor's detection threshold, were excluded. Hourly and daily averages were calculated for each participant."

Reviewer comments

4. Environmental covariates were missing. Factors such as temperature and humidity that may affect lung function were not controlled for, and it is recommended that this limitation be clarified in the discussion and that sensitivity analyses be performed when possible.

Authors' response

We thank the reviewer for this insightful comment. We agree that environmental factors such as temperature and humidity can influence lung function and should ideally be included as covariates. However, as these data were not collected in parallel with pollutant measurements in this feasibility study, sensitivity analyses including these parameters could not be performed. This limitation has been explicitly acknowledged in the Discussion section, and a cautionary statement has been added:

We have also emphasized the need for larger-scale studies integrating environmental parameters to better assess co-exposure and environmental confounding effects.

"The results should be interpreted with caution and mainly as indicative trends that justify larger-scale studies in the future. Environmental factors such as temperature and humidity, which can influence lung function, were not available for adjustment (34,35). The focus on $PM_{2.5}$ *alone, without accounting for co-pollutants, restricts assessment of co-exposure effects."*

Reviewer comments
5. The discussion section could be deepened. It is recommended that the discussion on the public health implications of the results and policy recommendations be strengthened, in particular the urgency of protective measures for children in high-exposure environments.

Authors' response
We thank the reviewer for this valuable suggestion. We have expanded the Discussion section to better highlight the public health implications of our findings and the need for protective measures for children exposed to high levels of air pollution.

Baseline spirometry data showed FEV_1 reversibility suggestive of presumptive asthma in 13.8% of participants, along with respiratory symptoms such as nocturnal cough, indicating possible obstructive patterns. Such early functional and symptomatic impairments, if persistent over time, may evolve into chronic expiratory flow limitation and increase the risk of early-onset chronic obstructive pulmonary disease (COPD). These findings emphasize the importance of early detection, health surveillance, and preventive actions to reduce children's exposure to particulate matter in their daily environments.

The following paragraph was added to the Discussion:

"The coexistence of respiratory symptoms and the reversibility of FEV1 observed on baseline spirometric data indicates functional impairment that remain amenable to targeted public health measures. Over time, persistent obstructive patterns in childhood may evolve into chronic expiratory flow limitation and increase the risk of chronic obstructive pulmonary

disease in adulthood (36,37). This underscores the need to monitor and reduce exposure levels among children living in such environments."

Reviewer comments

6. The authors mentioned that "High PM2.5 exposure and individual variability in lung function highlight the need for further research respiratory effects of air pollution in children", but this conclusion is obtained from the participants' current observation. Some recent studies indicate that early-life exposure to air pollution especially traffic-related air pollution (TRAP) including PM2.5 and NO2 is associated with childhood respiratory diseases (e.g., pneumonia and COVID-19). Some other new studies explain the potential mechanisms underlying the associations between TRAP (e.g., NO2) and allergic asthma, such as oxidative stress (e.g., ROS) and airway inflammation. I suggestion the authors should add some evidence in the effects of air pollution exposure on lung function. Please discuss your findings with (some of) these new articles to further support your results and explain the potential mechanisms.

Authors' response

We thank the reviewer for this helpful comment. In response, we have expanded the Discussion section to integrate recent evidence on the effects of air pollution, particularly fine particulate matter ($PM_{2.5}$), on childhood respiratory health. We have added new references, including a 2022 literature review that specifically addresses the relationship between particulate matter exposure and lung function in children (Zhang et al., 2022, DOI: 10.1016/j.envpol.2022.119735), as well as additional studies on the physiological mechanisms linking particulate matter to obstructive airway diseases and long-term respiratory consequences (Caillaud et al., 2019, DOI: 10.1016/j.rmr.2019.10.004).

"Obstructive patterns revealed at baseline spirometry, particularly those with reversibility, points to active inflammation and bronchial hyperresponsiveness, likely driven by chronic irritation from, combustion-related toxic compounds and fine particulate matter (34–35). The coexistence of respiratory symptoms […].

Reviewer 2

We thank Reviewer 2 for the interest and time accorded to the revision of our article. We addressed their questions and comments in a point-by-point response below.

In addition, we have taken this opportunity for edits to improve the writing that are in track change in the manuscript. We hope that this has also contributed to improve the overall quality of our manuscript.

Reviewer comments

I would however like the authors to evaluate if there is any empirical support for non-linear associations between ppFEV1 and $PM_{2.5}$.

Authors' response

We thank the reviewer for this comment regarding the possibility of a non-linear relationship between $PM_{2.5}$ exposure and ppFEV1. As suggested, we examined the literature using methodological approaches similar to ours in order to evaluate whether empirical evidence supports such non-linearity.

In the study by Delfino et al. (2008), “Personal and Ambient Air Pollution Exposures and Lung Function Decrements in Children with Asthma”, which also applied distributed lag models—the same analytical framework used in our study the authors reported that the association between %FEV1 and $PM_{2.5}$ concentrations was non-linear. Specifically, Figure 1 shows a non-linear exposure–response curve modeled using a 5th degree polynomial, demonstrating that the effect of PM2.5 on %FEV1 varies in a complex fashion across exposure levels and lag structure. These findings provide clear evidence of potential non-linearity in the $PM_{2.5}$ lung function relationship.

https://doi.org/10.1289/ehp.10911

Furthermore, a more recent study by De Rosa et al. (2022), “Associations between early-life exposure to PM2.5 and reductions in childhood lung function in two North American longitudinal pregnancy cohort studies”, also employed Distributed Lag Nonlinear Models (DLNM) to investigate the association between air pollutant exposures and several spirometric parameters. Their results likewise support the presence of non-linear effects of PM2.5 exposure on lung function during childhood.

Together, these studies, which rely on similar analytical frameworks to ours, provide strong empirical support for the possibility of non-linear associations between $PM_{2.5}$ exposure and ppFEV1.

DOI: 10.1097/EE9.0000000000000234

Reviewer comments

There is no mention of whether this was assessed. Also, the assessment of other model assumptions is lacking. Was for instance the assumptions of constant variance reasonably fulfilled ?

Authors' response

Thank you for this important comment. We agree that verifying model assumptions is essential, and we have conducted a series of diagnostic checks to ensure that the assumptions underlying the mixed-effects models with AR(1) structure were reasonably met.
For each model, we evaluated the normality of residuals using both visual inspection via Q–Q plots and statistical testing. As shown in the QQ plots, the points aligned closely with the reference line, indicating approximate normality of residuals. This was further supported by the Shapiro–Wilk test ($p > 0.05$), suggesting no significant deviation from normality.

R code (exemple for model_ar1) :

```
qqnorm(resid(model_ar1))
qqline(resid(model_ar1), col = "red")
shapiro.test(resid(model_ar1))
```

We also checked the assumption of homoscedasticity using both visual and statistical approaches. The residuals-versus-fitted plot showed that residuals were randomly distributed around zero with stable variance across the range of fitted values, suggesting no evidence of heteroscedasticity. We additionally performed a Breusch–Pagan test, which yielded p-values > 0.05, confirming that variance was constant.

R code (exemple for model_ar1) :

```
plot(fitted(model_ar1), resid(model_ar1))
abline(h = 0, col = "red")
library(lmtest)
bptest(lm(resid(model_ar1) ~ fitted(model_ar1)))
```

Given the longitudinal structure of the data, we evaluated autocorrelation of residuals, which is crucial when time dependency is modeled. Examination of the autocorrelation function (ACF) of residuals showed no significant peaks beyond lag 1, indicating that the AR(1) correlation structure adequately accounted for temporal dependence.

R code (exemple for model_ar1) :

```
acf(resid(model_ar1))
```

We also assessed multicollinearity using the Variance Inflation Factor (VIF) calculated on the fixed-effect variables. All VIF values were well below conventional thresholds (VIF < 5), suggesting that collinearity was not a concern.

Finally, random effects distributions were inspected to confirm their normality and adequate variability across subjects.

I added a short sentence to the method to ensure that we validated our models in the statistical analysis section:

"Model assumptions were evaluated by inspecting diagnostic residual plots and conducting statistical tests for normality, homoscedasticity, and autocorrelation."

No evidence of non-constant variance, residual autocorrelation beyond the AR(1) structure, or problematic multicollinearity was detected, indicating that the model assumptions were reasonably satisfied.

Reviewer comments

This seems to be a gross misinterpretation of the result provided by the model: *"Linking ppFEV$_1$ with PM$_{2.5}$ exposure revealed a transient decline in lung function, with the strongest effect observed few hours after an increase in PM$_{2.5}$ levels equivalent to the interquartile range of daily exposure."*

Authors' response

We thank the reviewer for this important comment. We agree that the term "strongest" was not appropriate and may have overstated the effect size. Given both the absence of certain covariates in our models and the relatively small variation in ppFEV$_1$ associated with PM$_{2.5}$ exposure (approximately 2%), we have revised the sentence to provide a more cautious interpretation.

The revised sentence in the Discussion now reads:

"Linking ppFEV$_1$ with PM$_{2.5}$ exposure revealed a transient decline in lung function, with a significant but small effect observed a few hours after an increase in PM$_{2.5}$ levels equivalent to the interquartile range of daily exposure."

We believe this revision more accurately reflects the modest effect observed and addresses the reviewer's concern.

VERSION 2 - REVIEW

**Reviewer**	**1**
**Name**	**Lu, Chan**
**Affiliation**	**Central South University, XiangYa School of Public Health**

Date **23-Dec-2025**

COI

Many thanks for the authors' careful explanations and revision. The authors have addressed all my comments. Now, I have no further comments.

Reviewer **2**

Name **Sommar, Johan**

Affiliation **Umea University Department of Public Health and Clinical Medicine, Sustainable health**

Date **08-Jan-2026**

COI

The authors have provided well motivated justifications to all my comments
